# Application of Consensus Scoring and Principal Component Analysis for Virtual Screening against β-Secretase (BACE-1)

**DOI:** 10.1371/journal.pone.0038086

**Published:** 2012-06-11

**Authors:** Shu Liu, Rao Fu, Li-Hua Zhou, Sheng-Ping Chen

**Affiliations:** 1 Department of Anatomy, Zhong Shan School of Medicine, Sun Yat-Sen University, Guangzhou, People’s Republic of China; 2 Guangdong Province Key Laboratory of Functional Molecules in Oceanic Microorganism, Zhong Shan School of Medicine, Sun Yat-Sen University, Guangzhou, People’s Republic of China; Russian Academy of Sciences, Institute for Biological Instrumentation, Russian Federation

## Abstract

**Background:**

In order to identify novel chemical classes of β-secretase (BACE-1) inhibitors, an alternative scoring protocol, Principal Component Analysis (PCA), was proposed to summarize most of the information from the original scoring functions and re-rank the results from the virtual screening against BACE-1.

**Method:**

Given a training set (50 BACE-1 inhibitors and 9950 inactive diverse compounds), three rank-based virtual screening methods, individual scoring, conventional consensus scoring and PCA, were judged by the hit number in the top 1% of the ranked list. The docking poses were generated by Surflex, five scoring functions (Surflex_Score, D_Score, G_Score, ChemScore, and PMF_Score) were used for pose extraction. For each pose group, twelve scoring functions (Surflex_Score, D_Score, G_Score, ChemScore, PMF_Score, LigScore1, LigScore2, PLP1, PLP2, jain, Ludi_1, and Ludi_2) were used for the pose rank. For a test set, 113,228 chemical compounds (Sigma-Aldrich® corporate chemical directory) were docked by Surflex, then ranked by the same three ranking methods motioned above to select the potential active compounds for experimental test.

**Results:**

For the training set, the PCA approach yielded consistently superior rankings compared to conventional consensus scoring and single scoring. For the test set, the top 20 compounds according to conventional consensus scoring were experimentally tested, no inhibitor was found. Then, we relied on PCA scoring protocol to test another different top 20 compounds and two low micromolar inhibitors (S450588 and 276065) were emerged through the BACE-1 fluorescence resonance energy transfer (FRET) assay.

**Conclusion:**

The PCA method extends the conventional consensus scoring in a quantitative statistical manner and would appear to have considerable potential for chemical screening applications.

## Introduction

Molecular docking-based virtual screening is widely used to discover novel ligands in the early stages of drug development [1], [2], [3], [4]. Various docking programs, such as DOCK [5], AutoDock [6], Surflex [7], FlexX [8], GOLD [9], and Glide [10], [11], have been developed. As an essential component of these programs, the scoring function is able to evaluate the fitness between the ligand and receptor guiding the conformational and orientational search of ligand-binding poses. Since the 1990s, several dozens of scoring functions have been reported in the literature [12], [13]. Current scoring functions can be roughly classified as force-field-based methods [5], [14], [15], empirical scoring functions [16], [17], and knowledge-based statistical potentials [18]. The existing limitations in current docking and scoring include a lack of protein flexibility, inadequate treatment of solvation, and the simplistic nature of the energy function employed [19], [20], [21], [22]. In particular, the major weakness of docking programs lies in the scoring functions [12], [13]. Considering the computational cost and time required for virtual screening, all of the current scoring functions use various approximations resulting in inaccuracy in the score and rank of the ligand-binding poses [19] as well as in false positives mixed in with the top scorers in the ranking list when virtual screening was performed with only a single scoring function. Some studies focus on calculating protein-ligand free binding energy, free energy perturbation (FEP), thermodynamic integration (TI) [23], [24], [25], MM-PB/SA, MM-GB/SA [26], [27], [28] and linear interaction energy (LIE) [29], [30], [31], which were used to perform post-docking processing. Although these methods are reported to be significantly more robust and more accurate than scoring functions, the accuracy is less than that usually required in typical lead optimization applications to differentiate highly similar compounds.

Attempts have been made to reduce the weakness of a single scoring function. In 1999, Charifson et al. introduced a consensus scoring method [20]. Many studies have suggested that employing consensus-scoring approaches can improve the performance by compensating for the deficiencies of the scoring functions with each other [19], [20], [21], [22]. Although the rationale for consensus scoring is still a subject of study, it has become a popular practice. Compared with the calculation of free binding energy mentioned above, the combination of three or four individual functions to perform consensus scoring is a relatively cheap computational method. Wang et al. carried out an idealized computer experiment with three different ranking strategies (“rank-by-number”, “rank-by-rank”, and “rank-by-vote”) to explore why the consensus scoring method performs better than the single scoring function [32]. However, the application of consensus scoring approaches is not always practical under ideal conditions because many obstacles prevent us from obtaining satisfied enrichment rates. These obstacles are as follows: (1) the binding scores calculated by the different scoring functions are typically given in different units and signs; (2) the scoring functions employed in consensus scoring often come from different categories; and (3) the linear relationship between many scoring functions (i.e., one scoring function can be expressed linearly by one or some other scoring functions).

In addition to the three ranking strategies introduced by Wang et al., several groups employed another consensus scoring method involving the linear combination of several scoring functions. In the study by Guo et al., five commercially available scoring function were weighted and summed to build a consensus score [33] by training with a 53-molecule set. Verdonk et al. also employed a linear combination of three scoring functions to re-rank the compounds [34]. Although an improvement was found for this consensus scoring method, the correlation between the scoring function and the experimental binding affinity is relatively poor. For a quantitative linear combination of the original scoring functions, the method for determining the appropriate weighting factors (correlation coefficients) for each scoring function is a complex problem.

In this study, we present an alternative method, principal component analysis (PCA) [35], [36], [37], for performing a linear combination of multiple scoring functions, formulating a modified ranking score and PCscore, and re-scoring and re-ranking the compounds after virtual screening. PCA is a powerful tool for pattern recognition, classification, modeling, and other aspects of data evaluation [36]. In addition, PCA is a linear transformation technique used to simplify a data set by reducing the dimensionality of multivariate data while preserving as much of the relevant information as possible. The principal components (PCs) are linear combinations of the original variables. The linear coefficients of the inverse relationships of linear combinations are called the component loadings. It represents the correlation coefficients between the original variables and the PC. In the present study, the first principal component (PC1) accounts for the maximum variance (eigenvalue) in the original dataset. The second principal component (PC2) is orthogonal (uncorrelated) to the first one, and it accounts for most of the remaining variance. This procedure is continued until the total variance is accounted for. The method of PCA makes use of intercorrelations that originate from the covariance matrix of the variables.

This work was performed as part of a project aimed at identifying strong, selective inhibitors of β-secretase (BACE-1) to overcome the shortcomings of the existing drugs to treat Alzheimer’s disease (AD) [38], [39], [40]. It is generally accepted that Alzheimer’s disease is caused by extracellular senile plaque deposition and that the intracellular formation of neurofibrillary tangles in the brain. β-amyloid peptides, which form the senile plaques, are formed by the action of the β-secretase and γ-secretase enzyme on the amyloid precursor protein (APP) [41], [42], [43]. The design of a lead compound that can inhibit APP binding to the active site of BACE-1 will prevent the cleavage of APP from the β-amyloid peptide and thus eventually prevent senile plaque formation [44], [45]. In the present study, the training set is composed of 50 confirmed BACE-1 inhibitors and 9950 inactive compounds [46], [47], [48]. Three rank-based virtual screening methods, individual scoring, conventional consensus scoring and PCA scoring were examined to identify BACE-1 inhibitors. To validate the efficacy of PCA ranking method, after virtual screening of 113,228 compounds (Sigma-Aldrich® corporate chemical directory) [49] and the BACE-1 fluorescence resonance energy transfer (FRET) assay, we found two drug-like and low-micromolar inhibitors.

## Methods

### 1. Preparation of the Screening Library

For the training set, in order to reduce artificial enrichment [34], a subset of WDI (World Drug Index) was specifically designated as inactive molecules. Firstly, WDI was filtered to eliminate compounds whose molecular weight was either less than 200 or greater than 800. In addition, the compounds whose log P is larger than 7 and the number of rotatable bonds is more than 15 should be abandoned. Secondly, the remaining 37,843 WDI compounds were subjected to diverse selection based on 2D UNITY fingerprints. The dissimilarity selection was performed by the Selector module in SYBYL, which resulted in 9950 compounds with a maximum Tanimoto index of 0.69. The active set was compiled from a diverse selection of 50 BACE-1 inhibitors from the total compounds available in the Prous Integrity Drugs & Biologics database [50]. This library of 10,000 compounds as a training set has an active content of 0.5%, which mimics real-life screening situations.

In order to extend the application of the present study, a total of 113,228 compounds (Sigma-Aldrich® corporate chemical directory, Z272000, 1997) [49] were used as the test set. Both the training set and test set compounds were stored as a SYBYL SLN list and converted to SYBYL mol2 format using Concord [51].

### 2. Preparation of the Protein Structure

The ligand-bound (1W51) structure of BACE-1 was used [52]. The procedure used to prepare the structure was as follows: hydrogen was added, the protonation states were assigned, and a highly limited optimization was performed to reduce bad contacts and the overall strain energy in the protein structure. The aspartate located on the active site was adjusted to an ideal protonation state, the Asp32 was protonated, the Asp228 was ionized [46].

### 3. Docking and Scoring

Virtual screening experiments were performed using the Surflex docking program [7], [53], [54] with an empirical scoring function (based on the Hammerhead docking system). The empirical scoring function has been updated and re-parameterized with additional negative training data along with a search engine that relies on a surface-based molecular similarity method. Standard parameters were used as implemented in the SYBYL software (version 8.1) [51]. The search strategy of Surflex employs an idealized ligand (called protomol), which utilizes various molecular fragments. Molecular fragments were tessellated in the active site and optimized based on the scoring function. The search algorithm utilized the morphological similarity function, which is evaluated between the protomol and the putative ligands. For the docking algorithms, a post-dock minimization procedure was applied using the BFGS quasi-Newton method and an internal Dreiding force field. For each compound, the top 30 ranked poses were saved.

Five scoring functions in SYBYL, including D_Score [55], G_Score [9], ChemScore [56], Surflex_Score [7], [53], and PMF_Score [57], [58], were applied to extract the stored poses. Next, five pose groups were produced, with each pose group containing 10,000 compounds.

For pose ranking, we use 12 scoring functions including the five scoring functions (D_Score, G_Score, ChemScore, Surflex_Score, and PMF_Score) from the SYBYL software and the seven scoring functions (LigScore1 [17], LigScore2 [17], PLP1 [59], PLP2 [59], jain [60], Ludi_1, and Ludi_2 [61], [62]) from the Discovery Studio software (version 2.1) [63]. Although the five pose groups generated by Surflex have been post-minimized using the internal Dreiding force field, these five pose groups were further minimized in the protein environment using the CFF force field [64] when the seven scoring functions were used for scoring by the Discovery Studio software.

All high-throughput docking calculations were performed on a Linux cluster using the CentOS 5.4 operating system.

### 4. Consensus Ranking

In this study, we adopted the “rank-by-number” strategy in the consensus scoring to combine the results of multiple scoring functions. The “rank-by-number” strategy was previously found to outperform the “rank-by-rank” and “rank-by-vote” strategy because it can summarize most of the information [32]. For the “rank-by-number” strategy, the consensus score of each binding pose is an average of the values determined by each of the individual scoring functions in a given consensus scoring scheme. With this strategy, a moderate number of scoring functions (i.e., three or four) have been proposed to be sufficient for significantly improving the results. Therefore, we chose 4 of the 12 scoring functions (D_score, jain, and Ludi_1, Surflex_Score) to perform consensus scoring in the present study.

Because the binding scores calculated by the different scoring functions are typically given in different units, it is almost impossible to compute consensus scores simply by summing up the binding scores determined by each of the individual scoring functions. Therefore, we scaled the binding scores of each scoring function to unit variance and centered (i.e., the mean value is zero, the standard deviation is one). The Z-scaled scoring function values (ZScore) are computed by
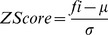
where f_i_ is the scoring value of a certain scoring function, μ is the mean value and σ is the standard deviation of this scoring function observed for the entire test set. The consensus score in a certain pose group is the average of ZScore by 4 individual scoring functions mentioned above in the given consensus-scoring scheme.

### 5. Principal Component Analysis of the Scoring Results

We describe PCA mathematically as described below.

Consider p random variables X_1_, X_2_, …, X_p_, the original system can be rotated to form a new coordinate. Let Σ be the covariance matrix associated with the random vector X′ = [X_1_, X_2_, …, X_p_]. The corresponding eigenvalue-eigenvector pairs are (λ_1_, e_1_), (λ_2_, e_2_), …, (λ_p_, e_p_), and the *ith* principal component is given by:
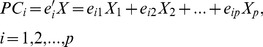
(1)


Then
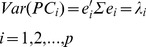
(2)


Thus, the principal components are uncorrelated, and their variances are equal to the eigenvalues of Σ.

Another property of the principal components is:
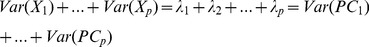
(3)Then the proportion of the total population variance due to the *kth* PC is:



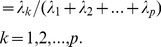
(4)Consequently, if most of the total population variance for large p can be attributed to the first two or three components, then these first two or three components could serve as a substitute for the original variables with a minimal loss of information. Moreover, if the weight of the last PCs occupied a highly trivial part of the total population variance, then the last PCs can be neglected (i.e., set to zero).

In the present study, there were five extracted pose groups, and we used only eight scoring functions, which included LigScore1, PLP1, jain, Ludi_1, D_Score, G_Score, ChemScore, and Surflex_Score, to perform the PCA for each group (The details are mentioned in the results section). Thus, for the training set, the eight scoring functions were used as the variables (i.e., columns of the matrix) and 10000 compounds were arranged in the rows of the matrix. Then the 10000×8 correlation matrix was established. Because the scoring functions in our test produce binding scores with different units and signs, the signs of the binding scores produced by LigScore1, PLP1, jain, Ludi_1, D_Score, G_Score, and ChemScore were reversed to ensure that positive binding scores always indicated higher binding affinities. All of the binding scores were scaled to unit variance and centered. Thus, each column of data had an average of zero and a standard deviation of one.

For each of the five scoring function extracted poses, we have calculated the eigenvalues and cumulative contribution rate. The first three principal components were extracted. Each principal component is a linear combination of eight Z-scaled scoring functions, which formulate a modified ranking score function, PCscore. PCscore is set to re-score and re-rank the extracted poses from each of the five scoring functions. PCscore can be written as follows:
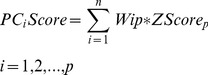
(5)


Where the n terms, ZScore_p_, are the Z-scaled scoring function, and the coefficients, w_ip_, are the loading values (i.e., the elements of p principal component eigenvector *e_p_*). For example, for PC1score
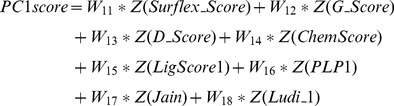
(6)The linear coefficient values (loading values) for 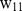, 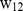 … 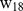 were the elements of the first principal component eigenvector, 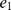.

In the present study, an SPSS version 16.0 statistical analysis package (SPSS Inc.) was used to normalize and calculate the principal components for all of the scoring data.

### 6. BACE-1 Enzymatic Assay

After virtual screening of the test set, 40 chemical compounds (Sigma-Aldrich Co. LLC) were purchased for experimental test through fluorescence resonance energy transfer (FRET) assay based on the results of conventional consensus scoring and PCA scoring. According to the previously described method [65], during the assay, compounds were diluted to 8 different concentrations and incubated 60 minutes at room temperature. Additional measurements were performed in the presence of detergent or with an incubation time of only 3 min to check for nonspecific effects (e.g., compound aggregation [66], [67]). Briefly, fluorescence progress curves of 30 µL reaction volumes were measured on a Gen5™ ELISA reader (BioTek® Instruments, Inc.) upon excitation at 545 nm and emission at 580 nm in 384-well microtiter plates (Corning, 3654). Linear regression analysis was calculated with the SPSS 16.0 software.

## Results

### 1. Individual Performance of Scoring Functions

Upon docking 10,000 compounds of the training set with Surflex, every compound yields 30 poses in the active pocket of the target (1W51), and no solution was found on the outside of the active pocket. After docking, we used five scoring functions to extract the pose and twelve scoring functions to rank the extracted poses resulting in 60 different scoring combinations. The top 1% of the ranked database was set as the threshold value, that is, the evaluation of the effectiveness of the scoring protocols involved numbering the actives for the top 100 candidates. The enrichment rates of the scoring protocols are presented in Table 1.

**Table 1:** Numbers of BACE-1 Inhibitors Retrieved in the Top 1% of the Ranked Database.

Pose extracted	Pose ranked											
	Surflex_Score	G_Score	D_Score	ChemScore	PMF_Score	LigScore1	LigScore2	PLP1	PLP2	jain	Ludi_1	Ludi_2
Surflex_Score	16	19	20	13	2	16	16	15	15	20	18	12
G_Score	17	17	15	11	2	12	12	9	9	17	18	13
D_Score	16	15	14	10	4	11	11	10	9	15	20	17
ChemScore	19	13	14	14	2	15	15	14	14	15	20	13
PMF_Score	14	16	19	8	6	16	16	15	15	14	15	10

The best-scored pose was always used to represent the plausible binding mode of a particular compound. In many cases, the pose varied from one to another as dictated by the separate scoring functions. Inspection of each scoring function in Table 1 indicated that the quality of the extracted poses is similar. No single scoring function outperforms the others with respect to the extraction. The Surflex_Score provided reliable poses that were ranked best by D_Score and jain. The D_Score and ChemScore provided reliable poses that were ranked best by Ludi_1.

For the pose ranking, it appears that the ranking by Ludi_1 retrieved more actives than the other scoring functions. Ludi_1 retrieved 20 inhibitors with D_Score and ChemScore pose extraction and 18 inhibitors with Surflex_Score and G_Score pose extraction. Ludi_1 was derived by empirically fitting a set of protein-ligand complexes with experimentally measured binding affinities. It is a sum of the five contributions including hydrogen bonds, perturbed ionic interactions, lipophilic interactions, the freezing of internal degrees of freedom of the ligand, and the loss of translational and rotational entropy of the ligand.

At the same time, D_Score also performs well for ranking the docking poses. It retrieved 20 inhibitors with Surflex_Score and 19 inhibitors with PMF_Score pose extraction. This good performance can be attributed to D_Score providing the most accurate approximation of the binding energy where both the electrostatic and hydrophobic contributions to the binding energy are counted. In addition, a distance-dependent dielectric attenuates the charge-charge, and other polar interactions were considered.

PMF_Score also provided reliable poses that were ranked best by D_Score. It yields 19 actives in the top 1% of the ranked list. However, it failed to rank any sensible docking poses regardless of what poses were extracted by itself or by the other scoring functions. Thus, for the BACE-1 target, PMF_Score appears to be more capable of accurately docking and correctly identifying the true binding mode, but the disadvantage of PMF_Score is the enrichment of active compounds.

Inspection of Table 1 demonstrates that two paired scoring with LigScore1 & LigScore2 and PLP1 & PLP2 retrieved an equal number of active compounds. It is not surprising that both Ligscore1 & Ligscore and PLP1 & PLP2 use the same scoring functions with only slightly different algorithms and parameters sets [68].

There are three different versions of Ludi (i.e., Ludi_1, Ludi_2, Ludi_3) [61], [62], [69]. According to the Discovery Studio user manual, only the weight factors employed by Ludi_2 for each term are derived by fitting to experimentally determined binding affinities. In fact, all three versions were tested for enrichment rates of virtual screening against BACE-1 in our study, and we found that Ludi_1 outperforms the other two versions.

### 2. Correlation Matrix

Prior to re-ranking the results from the virtual screening using consensus scoring and PCA, the intercorrelations between the scoring functions mentioned above were investigated. The original data of each scoring function were scaled to unit variance and centered. The correlations between the binding scores computed by the 12 scoring functions are summarized in Table 2.

**Table 2:** The Correlation Matrix of each scoring function (poses extracted by Surflex_Score).

	Z(Surflex_score)	Z(G_SCORE)	Z(PMF_SCORE)	Z(D_SCORE)	Z(CHEMSCORE)	Z(LigScore1)	Z(LigScore2)	Z(PLP1)	Z(PLP2)	Z(Jain)	Z(Ludi_1)	Z(Ludi_2)
Z(Surflex_score)	1											
Z (G_Score)	**0.639**	1										
Z(PMF_Score)	0.159	0.113	1									
Z(D_Score)	**0.689**	**0.771**	0.16	1								
Z(CHEMSCORE)	0.488	0.581	0.316	**0.636**	1							
Z(LigScore1)	0.423	0.527	0.133	**0.635**	0.518	1						
Z(LigScore2)	0.424	0.528	0.136	**0.635**	0.518	**0.999**	1					
Z (PLP1)	0.422	0.509	0.159	**0.632**	0.483	**0.972**	**0.975**	1				
Z (PLP2)	0.418	0.509	0.153	**0.63**	0.486	**0.977**	**0.98**	**0.999**	1			
Z (Jain)	0.459	0.416	0.209	0.533	0.302	**0.614**	**0.616**	0.597	**0.603**	1		
Z (Ludi_1)	0.498	0.374	−0.051	0.331	0.026	0.134	0.134	0.101	0.103	0.386	1	
Z (Ludi_2)	0.326	0.188	−0.058	0.116	−0.152	−0.18	−0.179	−0.199	−0.198	0.172	**0.911**	1

Kaiser-Meyer-Olkin Measure of Sampling Adequacy = 0.818.

Bartlett’s Test of Sphericity :Approx. Chi-Square = 2.265E5.

Correlation coefficients >0.6 are marked in boldface type.

For the four scoring functions (i.e., Ligscore1, Ligscore2, PLP1 and PLP2), Table 2 exhibited a high correlation between any two of them. The correlation was higher for LigScore and PLP (R = 0.97∼0.98) because they belong to the empirical scoring function category and the sum of the pairwise linear potentials between the ligand and the protein heavy atoms with parameters is dependent on the interaction type.

In addition, Ludi_1 and Ludi_2 also exhibited a very high correlation (R = 0.911). However, the correlation coefficients between Ludi and the other functions, such as PLP and LigScore, were smaller because the master equations that describe the binding free energy used in Ludi are different from those used in the PLP and LigScore functions. In addition, the algorithms vary for the same term in the master equation, such as hydrogen bonding and hydrophobic effect.

Furthermore, there was a higher correlation between G_Score and D_Score (R = 0.771). We were not surprised by this result because both functions are in the same category, both of their algorithms adopted force-field-based methods that estimate the enthalpic contribution upon binding, and both of them use a very similar treatment of the energy terms.

As depicted in Table 2, moderate correlation was exhibited by Surflex_Score and either the D_Score or the G_Score function; between D_Score and the ChemScore, LigScore1, LigScore2, PLP1, or PLP2 function; and between Jain and the LigScore1, LigScore2, or PLP2 function. This is consistent with virtually all of the scoring functions being designed to reflect the basic features in protein-ligand interactions including hydrogen bonds and hydrophobic contacts. Moreover, the binding scores computed by these scoring functions are all correlated, to some extent, to the known binding constants. Therefore, some intercorrelation between them is natural.

PMF shared the least with all of the other scoring functions. Its unique knowledge-based algorithm parameterized using crystal complexes is different from the rest of the scoring functions being considered [57], [58].

### 3. Consensus Scoring

The hit-rates observed among the top 1% of the screening set using the “rank-by-number” strategy are shown in Table 3. By comparing the performance of all of the consensus ranking schemes tested, it appears that the consensus ranking does statistically outperform the best of the individual scoring function. The Surflex_Score pose extracted group produced 24 hits in the top 1% of the screening set when the quadruple-scoring scheme was applied. The improvements are not trivial. The best individual scoring function, jainScore, produces only 20 hits in the top 1% of the screening set.

**Table 3:** Enrichment Rates of Consensus Scoring Schemes in the “Rank-by-Number” Experiments (in the Top 1% of the Ranked list, Surflex_Score pose extracted group).

Scoring function	Enrichmentrate	Double scoring	Enrichmentrate	Triple scoring	Enrichmentrate	Quadruple scoring	Enrichmentrate
A	16	A+B	24	A+B+C	23	A+B+C+D	24
B	20	A+C	19	A+B+D	23		
C	20	A+D	18	A+C+D	22		
D	18	B+C	23	B+C+D	23		
		B+D	22				
		C+D	22				

a A  =  Surflex_Score; B  =  D_Score; C  =  jain; D  =  Ludi_1.

Our results are in agreement with the previous study, which suggested that, in theory, combining multiple scoring functions should always provide improved performance over individual scoring functions in simulated virtual screening experiments [32]. According to the present results, we cannot definitively conclude that more scoring functions result in a better performance. For example, application of double-scoring schemes (e.g., Surflex_Score&D_Score) could also obtain 24 hits in the top 1% of the screening set, which is the same result obtained using the quadruple-scoring scheme. However, it is important to note that double-scoring schemes do not outperform the best individual scoring function in all cases. For example, Surflex_Score&jain could obtain only 19 hits, which is slightly less than the 20 hits obtained from the single scoring function, jain. Therefore, it is largely unpredictable which combinations of scoring functions would produce the optimal results. In practice, it is better to test all possible combinations of scoring functions on the appropriate samples.

Some studies have shown that consensus ranking does not outperform the best individual scoring function [70], [71]. They argued that if one knew in advance which scoring functions worked best for a given target, the better performance could be achieved using this scoring function alone and by concentrating on only the highest ranking compounds. Given the contradiction between their arguments and our results, we explained as followed:

Firstly, the three scoring functions (D_Score, jain, Ludi_1) that we chose performed the best in single scoring. It is important to consider which scoring functions should be chosen to perform consensus scoring. We used an additional four scoring functions to perform consensus scoring, but the performance was not as good as the four functions that we chose. Due to the variation in the performance of the different scoring functions, blindly choosing scoring functions to perform consensus scoring will decrease the enrichment rates. Secondly, the four scoring functions that we chose were independent of each other. It is reasonable to expect that an effective consensus scoring scheme would combine complementary scoring functions rather than highly correlated ones. As indicated in Table 2, if the consensus scoring schemes contained Ligscore1 and Ligscore2 as well as PLP1 and PLP2, it would perform poorly compared to the other schemes. Thirdly, Verdonk et al. performed a computational experiment on the simulated effect of consensus ranking with an increasing number of scoring functions using the rank-by-number protocol [34]. They noted that if the first scoring function performs well (standard deviation = 1.0), then adding additional scoring functions (standard deviation = 3.0) to perform consensus ranking can reduce the enrichment rates compared to the most accurate single scoring function. The main reason for this phenomenon was that noise was added to the protocol. However, if all of the scoring functions have a standard deviation of 2.0, then adding extra scoring functions to the consensus ranking protocol always improves the enrichment rates. In our study, all of the binding scores were scaled to unit variance and centered, which was consistent with the results reported by Verdonk et al [34].

In summary, our present results suggest that application of triple-scoring and quadruple-scoring schemes are more robust and accurate than any single scoring procedure.

### 4. Principal Component Analysis

Principal component analysis (PCA) can extract information from large-scale scoring data and decompose multiple scoring functions into one or two scoring functions, which can be used to re-score and re-rank the compound binding poses. As mentioned above, the PLP1&PLP2, ligscore1&ligscore2 and Ludi_1&Ludi_2 have high relative between each other. In addition, PMF_Score failed to rank any sensible docking poses. Thus, we do not use these four scoring functions in the following study. We constructed five 10000×8 matrices using the remaining eight scoring functions as the matrix column and the 10000 compounds as the raws. Then the matrix was transformed such that each column of data had an average of zero and a standard deviation of one.

As observed from Table 4, we can derive eight uncorrelated descriptors (the principal components) from each scoring matrix. The weight of each principal component was determined based on their contribution rate to the variance (eigenvalues, λ). We found that the first three principal components (PC1, PC2, PC3) account for >80% of the total variance for each pose group. The PC4, PC5, PC6, PC7, and PC8 could be omitted in further studies due to their trivial contribution to the total variance. This result is in agreement with the aim of introducing PCA to significantly minimize the number of variables and to omit the principle components with low variance that will not affect the total variance.

**Table 4:** The eigenvalues and cumulative contribution rate of each five scoring function extracted poses.

Component	Pose extracted									
	Surflex_Score		G_Score		D_Score		ChemScore		PMF_Score	
	λ	% of Variance	λ	% of Variance	λ	% of Variance	λ	% of Variance	λ	% of Variance
PC1	4.551	56.885	4.425	55.316	4.402	55.029	4.466	55.824	4.506	56.322
PC2	1.25	15.623	1.218	15.221	1.163	14.533	1.187	14.839	1.229	15.361
PC3	0.924	11.556	0.931	11.631	0.923	11.542	0.888	11.095	0.876	10.95
PC4	0.405	5.059	0.538	6.721	0.56	6.995	0.571	7.135	0.487	6.084
PC5	0.334	4.17	0.411	5.139	0.41	5.119	0.395	4.943	0.38	4.756
PC6	0.315	3.938	0.308	3.846	0.312	3.896	0.287	3.585	0.301	3.764
PC7	0.197	2.457	0.15	1.871	0.205	2.557	0.184	2.295	0.201	2.511
PC8	0.025	0.313	0.02	0.254	0.026	0.329	0.023	0.286	0.02	0.252

These principal components may lack physical meaning by themselves because they may act as statistical descriptors. Nevertheless, we could still assess the physical meaning of each PC according to the energy terms of each scoring function. To the best of our knowledge, the physical meaning of PC1 could be attributed to van der Waals interactions, the physical meaning of PC2 could be attributed to electrostatic interactions, and the physical meaning of PC3 could be attributed to the hydrophobic interactions between the protein and ligands.

The loadings express how well the new abstract principal components correlate with the old variables. Loading values (i.e., correlation coefficients) >0.7 are marked in boldface type in Table 5. For the first new abstract principal component, PC1 accounts for approximately 56% of the total variance. All of the original scoring functions have a positive correlation with PC1. The loading values of the eight original variables in PC1 were small and approximately equal to each other, which means none of them plays a dominant role in the explanation of PC1, i.e., for van der Waals interactions.

**Table 5:** Loading values (i.e., correlation coefficients).

Pose extracted	Pose ranked														
	Surflex_Score			G_Score			D_Score			CHEM_Score			PMF_Score		
Loading value	PC1	PC2	PC3	PC1	PC2	PC3	PC1	PC2	PC3	PC1	PC2	PC3	PC1	PC2	PC3
Surflex_Score	0.356	0.322	−0.182	0.276	0.484	−0.224	0.278	0.457	0.239	0.272	0.46	−0.197	0.311	0.379	−0.233
G_Score	0.38	0.14	−0.314	0.42	0.001	−0.217	0.413	−0.01	0.197	0.418	−0.018	−0.238	0.419	−0.007	−0.176
D_Score	0.416	0.05	−0.208	0.41	0.102	−0.295	0.391	0.064	0.351	0.387	0.084	−0.378	0.38	0.123	−0.359
ChemScore	0.325	−0.178	−0.549	0.318	−0.228	−0.575	0.322	−0.27	0.54	0.349	−0.225	−0.481	0.335	−0.208	−0.512
LigScore1	0.39	−0.337	0.274	0.407	−0.331	0.289	0.412	−0.296	−0.296	0.41	−0.296	0.296	0.41	−0.295	0.28
PLP1	0.384	−0.352	0.282	0.4	−0.348	0.299	0.405	−0.315	−0.304	0.402	−0.319	0.302	0.403	−0.318	0.292
Jain	0.334	0.034	0.53	0.4	0.073	0.508	0.338	0.093	−0.53	0.334	0.089	0.556	0.337	0.099	0.57
Ludi_1	0.193	0.635	0.299	0.198	**0.74**	0.229	0.219	**0.72**	−0.164	0.198	**0.73**	0.2	0.159	**0.775**	0.185
hit-rates*	26	9	0	21	8	0	21	5	0	24	9	0	26	10	0

* Numbers of BACE-1 inhibitors retrieved in the top 1% of the ranked database.

PC2 accounts for approximately 15% of the total variance. It negatively correlates with Ludi_1, ChemScore, LigScore_1, and PLP1. Among the eight loads in PC2, Ludi_1 exhibits the maximum value (0.64, 0.74, 0.72, 0.73, 0.78) for each of the five pose group. It indicates that Ludi_1 plays a dominant role in the explanation of PC2, i.e., electrostatic interactions. Therefore, this result demonstrated that the greatest contribution to the electrostatic interactions in the receptor-ligand originates from the ludi_1 function.

PC3 accounts for approximately 11% of the total variance. It is interesting to note that PC3 negatively correlates with Surflex_Score, D_Score, G_Score, and ChemScore (SYBYL software) but positively correlates with PLP1, LigScore1, jain, and Ludi_1 (Discovery Studio software).

**Figure 1:**
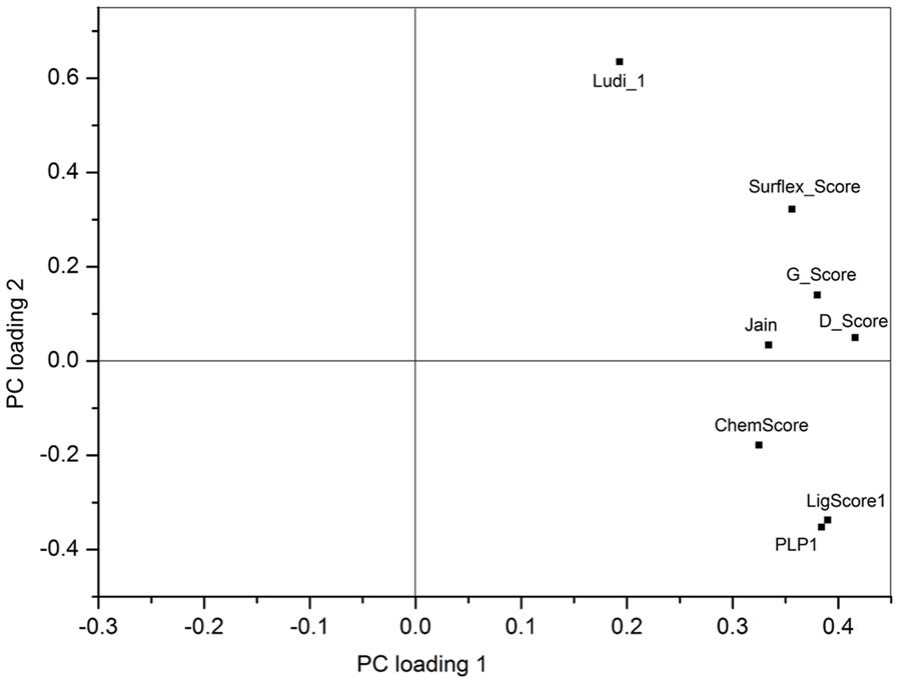
Principal component loadings. Loading 1 versus loading 2.

The first two PC loadings against each other are shown in Figure 1. Because the PCA is invariant to the mirroring through the origin, the data shown here indicate that there is a significant correlation between LigScore1 and PLP1. Likewise, the correlation between G_Score and D_Score in relation to the data is unambiguous and significant. There is no need to measure and evaluate all of the variables to achieve the same characterization in further studies. It is sufficient to measure one variable per group.

The present results show that Jain contains nearly the same information as D_Score and has low loading on PC2. Because PC2 could be attributed to electrostatic interactions between the protein and the ligands, Jain has no significant influence on the electrostatic interactions between the protein and the ligands. Among the eight loads in PC2, Ludi_1 exhibits the maximum value (Figure 1), which means Ludi_1 plays a significant role in the description of PC2.

According to Eq 5, the PC is a linear combination of multiple original variables. Therefore, we can formulate the first three PCscore scoring functions for each pose group from the data in Table 5. For example, for the poses extracted by Surflex_Score, the first PC scoring function was:
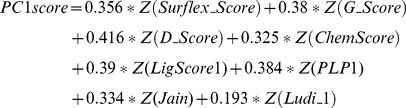
(7)While the second PC scoring function was:
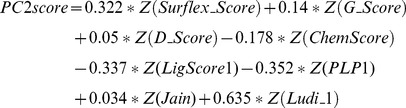
(8)Then the third PC scoring function was:
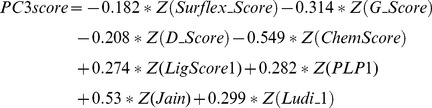
(9)Next, the docked compounds are re-scored and re-ranked using the PCscore scoring functions as mentioned above. The enrichment rates are also determined by noting the numbers of active compounds retrieved in the top 1% of the ranked database (Table 5). When comparing the enrichment rates from PC1score to the results obtained from a single scoring function or conventional consensus scoring functions, PC1score exhibited better performance for the enrichment rates regardless of the scoring function employed to extract the compound pose. For example, PC1score yields 26 active compounds in the Surflex_Score and PMF_Score pose group, which outperforms both the single scoring function with 20 or less active compounds and the consensus scoring method with 24 active compounds.

As indicated in Table 5, application of PC1score results in more active compounds than the application of PC2score and PC3score for each of the five pose group due to the descriptiveness of the first principal component, which shares the maximum amount of the whole variance followed by the decreasing descriptiveness of the other PCs. We did not obtain any active compounds in the top 1% of the ranked database using PC3score because the values of the eigenvalue of PC3 were <1.

**Figure 2:**
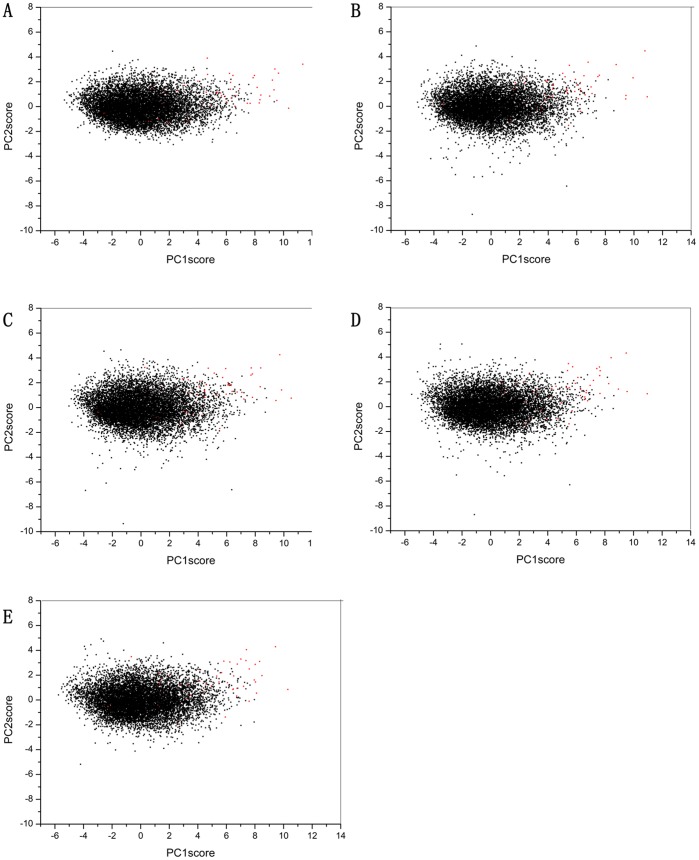
Two-dimensional score plot of the principal component analysis of the screening library set (50 known inhibitors and 9950 non-inhibitors) formed by the two most important principal component scores (PC1score versus PC2score) derived from the scoring data. Known inhibitors are depicted as red circles, non-inhibitors as black squares. **A**: Surflex_score extracted poses. **B**: D_Score extracted poses. **C**: G_Score extracted poses. **D**: ChemScore extracted poses. **E**: PMF_Score extracted poses.

The PCA can illustrate the relationship between the different compounds and the different scoring functions. The compounds can be plotted in the space defined by two PCs (score plot, Figure 2), which identifies active compounds as a function of inactive compounds. The values of the scores can be understood as the values of the compound in the new variable space, i.e., the principal component space. In Figure 2, active compounds are depicted as red circles, and inactive compounds are depicted as black squares. The results showed that most of the data were scattered along the PC1 axis. The scattering variation along the PC1 axis is larger than that along the PC2 axis, which corresponds to the values of eigenvalue and reflects the descriptive power of first two PCs scores.

Because the new variable space is normalized with zero mean, the most active compounds, which are farther from the origin, have values significantly different from the mean and can be considered outliers. Moreover, we found that the scattering positions of the true BACE-1 inhibitors are located on the right side of the PC1 axis indicating that PC1 plays a significant discriminating role among active and inactive compounds. As for the PC2 axis, all of the data were scattered in a narrow area from -3 to 3, and the discriminating power among active and inactive compounds was weaker.

### 5. BACE-1 Enzymatic Assay

To further investigate the validity of docking based virtual screening, after virtual screening of 113,228 compounds against BACE-1 by Surflex, we employed conventional consensus scoring and PCA scoring protocol to select compounds for experiment test against BACE-1. Standing the view of economic point, the number of compounds to be tested in computational docking studies should be restricted in a smaller and reasonable range, therefore, we used several filters to the select the final compounds in Surflex_score extracted pose for experiment.

In an initial attempt, we employed conventional consensus scoring protocol to select the potential inhibitors. Firstly, we selected the top 300 compounds according to the ranking results of the conventional consensus scoring protocol, e.g., quadruple-scoring scheme (Surflex_Score&D_Score&jain&Ludi_1); Secondly, visual inspection has been given to all individual complexes for the top 300 compounds, Surflex provides interaction information between the protein and ligand for each docking experiment, only those compounds with interactions to the catalytic residues (Asp32 and Asp228) and other relevant residues are extracted; Thirdly, to remove unsuitable compounds that would not reach and pass the clinical trials due to undesired and toxic properties, the so-called Lipinski “Rule-of-five” [72], a very popular method was used to evaluate the drug likeness of a candidate structure. Finally, 20 drug-like compounds were selected to purchase from Sigma-Aldrich Co. LLC. By the BACE-1 fluorescence resonance energy transfer (FRET) assay, disappointingly, no inhibitor was found among the compounds selected by the conventional consensus scoring protocol.

Based on the theory that PCA can summarize most of the information from the original scoring functions, we employed PC1score to re-rank the 113,228 Surflex_score extracted poses, as mentioned above, PC1score is a linear combination of eight scoring functions (Surflex_Score, G_Score, D_Score, ChemScore, LigScore1, PLP1, Jain and Ludi_1). By the same filter protocol as the conventional consensus scoring, another 20 drug-like compounds were select for purchase among the top 300 compounds. Excitingly, this time two compounds (S450588 and 276065), with a remarkable 10% hit rate, emerged as the BACE-1 inhibitors in the low-micromolar range, showing IC_50_ values of 51.6 and 85.3 µM, respectively (Table 6). The chemical structures of these two compounds were showed in Table S1.

**Figure 3:**
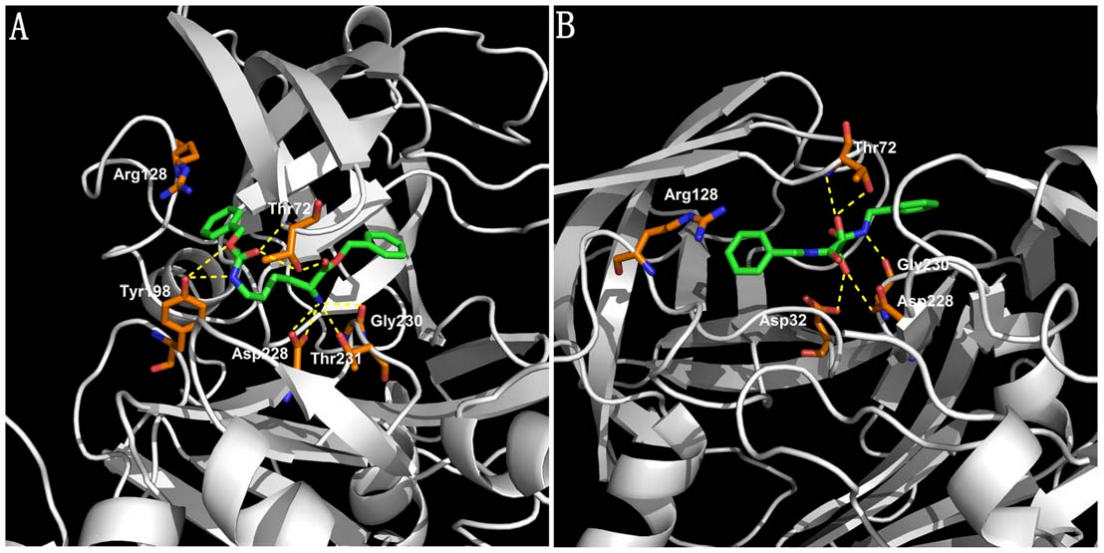
Binding modes of compounds in the BACE-1 catalytic site (PDB entry: 1W51). **A**: compound **1**. **B**: compound **2**. The compounds are rendered in green stick models, and the residues are rendered in orange sticks. Hydrogen bonds between compounds and residues are highlighted by yellow dashed lines. Pictures were generated with PyMOL (http://www.pymol.org).

As depicted in Figure 3A, after compound **1** docking into 1W51 structure, the protonated 2-NH_3_ group of the lysine moiety form hydrogen bond with Asp228, Gly230 and Thr231, respectively, the 6-NH group form hydrogen bond with Tyr198. The benzyl ring (P1) fills the S1 pocket shaped by the Tyr71, Phe108, and Trp115 residues, while carbobenzyloxy moiety (P2′) fills the S2′ pocket shaped by the Tyr71, R128, and Y198 residues, so as to allow the carbonyl group to form hydrogen bond with the Thr72 residue, the benzyl group to establish a cation-π interaction with the guanidine group of Arg128.

**Table 6:** Structures of Compounds Showing Inhibitory Activity against BACE-1.

**Molecule**	**ID**	**Name**	**MW (g mol^-1^)**	**IC_50_ (μM)**
**1**	S450588	N-6-Carbobenzyloxy-L-Lysine Benzyl Ester Hydrochloride	406.9	51.6
**2**	276065	(−)-N,N′-Dibenzyl-D-tartaric diamide	328.4	85.3

As depicted in Figure 3B, in the catalytic site, for the small size and symmetric overall shape of compound **2**, one of the hydroxyl group of the tartaric diamide core are involved in hydrogen bonds with the side chain of the catalytic Asp32 and Asp228, respectively, whereas the other hydroxyl group form hydrogen bond with Thr72, and one of the amide group form hydrogen bond with Gly230. Both sides of compound **2** are benzyl groups, one of the benzyl group occupy the S2 pocket shaped by the Asn233, Arg235 and Ser325 residues, the other benzyl group occupy the S2′ pocket, establish a cation-π interaction with the guanidine group of Arg128.

## Discussion

BACE-1 is one of the major Alzheimer’s disease target [38], [39], [40]. To find novel BACE-1 inhibitors, a lot of academic research centres and pharmaceutical industries are quite active in this field. Merck research group performed in vitro high-throughput screening (HTS) and found a single molecule (a 1,3,5 trisubstituted benzene) as a hit from a multi-million compound library [73]. Johnson and Johnson also reported a novel cyclic guanidine screening lead, the initial screening lead had an IC_50_ value of 900 nM [45]. Astex Therapeutics has taken a fragment-based lead generation approach [74]. After the virtual screening of a fragment library, a small number of potential structures were soaked with BACE-1 crystals in anticipation of obtaining a co-crystal with the enzyme. Huang et al. performed in silico Screening of 180,000 small chemicals, they found 10 diacylurea inhibitors showed an IC_50_ value lower than 100 µM in a enzymatic assay and four of them were cell penetrant (EC_50_<20 µM) [75].

Despite the availability of many reliable in silico approaches and robust in vitro commercially available assays, discovering BACE-1 inhibitors still remains a challenging task. In the present study, based on the virtual screening of 10,000 compounds of training set, the PCA approach yielded consistently superior rankings compared to conventional consensus scoring and single scoring. By virtual screening of 113,228 compounds, and application of PCA approach to re-rank the score list, two drug like BACE-1 inhibitors were emerged as an effective low-micromolar inhibitors. It suggested that the application of PCA provides a more robust strategy for ranking compounds. The advantages of PCA are as follows.

First, PCA is efficient. For each five pose group, the application of PCA can result in superior enrichment of known inhibitors compared to either the conventional consensus scoring or the best individual scoring. In addition, the application of PCA for post-processing of the scoring data from virtual screening was not time-consuming. Second, PCA is reliable. PCA is mainly useful when there is limited knowledge about the target and its inhibitors. If we have no idea which scoring function would return the best enrichment rates (i.e., several known active compounds are required to determine the best scoring functions), then adopting PCA to formulate a new scoring function can provide better performance than blindly using a scoring function or some combination scoring functions when performing virtual screening of an uncertain protein. Third, PCA is economical**.** The objectivity of PCA is due to the fact that it relies entirely on the input data itself instead of developing new scoring functions. In this study, we have employed only an alternative method to exploit the utility of present scoring functions. Bioactive information on active compounds, such as K_i_ and IC_50_, and training with a data set were not necessary.

When a training set was available, there are several other groups that perform a different type of post-docking processing using statistical methods and data mining. Wilton et al. discussed the use of several rank-based virtual screening methods, such as binary kernel discrimination, similarity searching, sub-structural analysis, support vector machine (SVM), and trend vector analysis, for prioritizing compounds in lead-discovery programs [76], [77]. Jacobsson et al. employed three different multivariate statistical methods including PLS discriminant analysis, rule-based methods, and Bayesian classification to analyze multidimensional scoring data from four different target proteins (i.e., the estrogen receptor R (ERR), matrix metalloprotease 3 (MMP3), factor Xa (fXa), and acetylcholine esterase (AChE)). The classifiers that they built showed that the precision is approximately 90% for three of the targets and approximately 25% for acetylcholine esterase for correctly predicting an active compound [78]. The difference between our work and their’s is that we do not need a training set because PCA is a form of unsupervised learning and relies entirely on the input data itself. In addition, PCA is simpler than the methods mentioned above (SVM, trend vector analysis, PLS discriminant analysis, rule-based methods, and Bayesian classification).

With eight different scoring functions, Terp et al. docked a set of known inhibitors to three different matrix metalloproteases. They obtained scores analyzed using PCA and partial least-squares methods (PLS) [79]. The regression model they built has a good q^2^ for predicting the activity of active compounds. The major difference between the present work and the work performed by Terp et al. is that we performed structure-based virtual screening on both active and inactive compounds. Terp et al. included only known inhibitors to quantitatively predict the binding affinity. They did not discuss whether the docking scores have been calculated from a docking mode of an inactive compound that does not actually bind. In the virtual screening process, more attention is focused on how to identify promiscuous active compounds in a database of mainly inactive compounds rather than on how to rank a set of known binders (i.e., predicting the binding affinity of the different active compounds).

It should be emphasized the necessity of experimental validation for potential researchers, because no ranking method may help if not associated with verification in the experiment. In an initial attempt, we applied the conventional consensus scoring method to re-rank the score list and experimental test through BACE-1 FRET assay, no inhibitor was found. However, when we applied the PCA scoring method and experimental test through BACE-1 FRET assay, a remarkable 10% hit rate was achieved. On this basis, we summed up some experience as followed: when virtual screening of a new chemical database, the potential researchers usually do not know which kind of individual scoring function work best for the target protein, furthermore, for consensus scoring protocol, they are uncertain which kind of scoring functions should be used to combine for getting the best enrichment rates. Once trapped in this dilemma, the researchers could use PCA scoring protocol to re-rank the results from the virtual screening, a prominent advantage of application of PCA scoring protocol can summarize most of the information from the original scoring functions and improve the enrichment rate, which has been proved to be robust and reliable in the present study.

In conclusion, although the PCA approach is not intended to improve all aspects of virtual screening, such as generating more accurate binding poses, it extends conventional consensus scoring in a quantitative statistical manner, therefore, it has great potential for use in the virtual screening process. Future experiments are needed to further analyze the performance of PCA for other receptor binding sites. We believe that the two low-micromolar inhibitors described here may represent a starting point for finding potent and selective molecules capable of preventing BACE-1 activity for the treatment of Alzheimer’s disease.

## Supporting Information

Table S1 Structures of Compounds Showing Inhibitory Activity against BACE-1.
(DOC)
