# Supplementary material for: Application of Consensus Scoring and Principal Component Analysis for Virtual Screening against β-Secretase (BACE-1)
Source: PLoS One. 2012 Jun 11;7(6):e38086. doi: 10.1371/journal.pone.0038086 (PMC3372491; doi:10.1371/journal.pone.0038086)
Supplement: Table S1 — Structures of Compounds Showing Inhibitory Activity against BACE-1. (DOC) [file pone.0038086.s001.doc]

**Table S1. Structures of Compounds Showing Inhibitory Activity against BACE-1**

| **Molecule** | **ID** | **Name** | **MW** (g mol^-1^) | **Structure** | **IC_50_** (μM) |
| --- | --- | --- | --- | --- | --- |
| **1** | S450588 | N-6-Carbobenzyloxy-L-Lysine Benzyl Ester Hydrochloride | 406.9 |  | 51.6 |
| **2** | 276065 | (−)-*N*,*N*′-Dibenzyl-D-tartaric diamide | 328.4 |  | 85.3 |
